# Carvedilol Ameliorates Early Diabetic Nephropathy in Streptozotocin-Induced Diabetic Rats

**DOI:** 10.1155/2014/105214

**Published:** 2014-06-04

**Authors:** Mohamed A. Morsy, Salwa A. Ibrahim, Entesar F. Amin, Maha Y. Kamel, Soha A. Abdelwahab, Magdy K. Hassan

**Affiliations:** ^1^Department of Pharmacology, Faculty of Medicine, Minia University, El-Minia 61511, Egypt; ^2^Department of Histology, Faculty of Medicine, Minia University, El-Minia 61511, Egypt; ^3^Department of Physiology, Faculty of Medicine, Minia University, El-Minia 61511, Egypt

## Abstract

Diabetic nephropathy results in end-stage renal disease. On the other hand, carvedilol has been reported to have various pharmacological properties. The aim of this study therefore is to evaluate the possible protective effect of carvedilol on streptozotocin-induced early diabetic nephropathy and various mechanisms underlie this effect in rats. Single i.p. injection of streptozotocin (65 mg/kg) was administered to induce early diabetic nephropathy in Wistar rats. Oral administration of carvedilol at a dose level of 1 and 10 mg/kg daily for 4 weeks resulted in nephroprotective effect as evident by significant decrease in serum creatinine level, urinary albumin/creatinine ratio, and kidney index as well as renal levels of malondialdehyde, nitric oxide, tumor necrosis factor-**α**, and cyclooxygenase-2 with a concurrent increase in creatinine clearance and renal reduced glutathione level compared to diabetic untreated rats. The protective effect of carvedilol was confirmed by renal histopathological examination. The electron microscopic examination indicated that carvedilol could effectively ameliorate glomerular basement membrane thickening and podocyte injury. In conclusion, carvedilol protects rats against streptozotocin-induced early diabetic nephropathy possibly, in part, through its antioxidant as well as anti-inflammatory activities, and ameliorating podocyte injury.

## 1. Introduction

Diabetic nephropathy, an important complication of both types of diabetes, is the most common cause of end-stage renal disease and occurs as a result of an interaction between metabolic and hemodynamic factors [1]. It is characterized histologically by definite changes including increased glomerular basement membrane thickening and clinically progressive albuminuria [2]. Podocytes, residing on the outer surface of the glomerular basement membrane, play a key role in maintaining the structure and function of the glomerular filtration barrier and they have an essential role in the development of proteinuria [3]. On the other hand, oxidative stress is considered as a key component in the development of diabetic complications including diabetic nephropathy [4]. Alternatively, inflammation is a major contributing factor in deterioration of kidney function due to diabetes [5].

Carvedilol was reported to act as a nonselective third-generation *β*-blocker as well as a selective *α*_1_-blocker. Moreover, the vasodilatory *β*-blocker carvedilol exhibits additional effects on ameliorating oxidative stress and inflammation [6, 7], rendering it an attractive candidate for the prevention of early diabetic nephropathy. Carvedilol has shown renal protective properties against gentamicin- [8] and cisplatin- [9] induced renal toxicities as well as renal ischemia-reperfusion injury [10]. Furthermore, diabetes mellitus and hypertension frequently coexist. Patients with both diabetes and hypertension are at high risk from the development of nephropathy. Therefore, carvedilol, an antihypertensive drug, could be important preventive therapeutic option for these patients. Thus, the aim of the present study was to evaluate the protective effect of carvedilol on streptozotocin- (STZ-) induced early diabetic nephropathy in rats and to reveal the mechanisms implicated in these protective effects.

## 2. Materials and Methods

### 2.1. Animals

Male Wistar rats weighing 150–180 g were used after one week for proper acclimatization to the animal house conditions (12 h lighting cycle and 25 ± 2°C temperature) and had free access to standard rodent chow and water. Procedures involving animals and their care were conducted in conformity with the protocols of the Research Advisory Ethical Committee of Faculty of Medicine, Minia University, Egypt, and the EEC Directive of 1986 (86/609/EEC).

### 2.2. Chemicals

Global Napi Pharmaceuticals (Giza, Egypt) generously provided carvedilol powder. STZ was purchased from Sigma-Aldrich Corp. (St. Louis, MO, USA). Antibody against cyclooxygenase-2 (COX-2) was purchased from Thermo Fisher Scientific Inc./Lab Vision (Fremont, CA, USA). All other chemicals were of analytical grade and were obtained from commercial sources.

### 2.3. Experimental Induction of Diabetes

Diabetes was induced in overnight fasted rats by i.p. injection of freshly prepared STZ (60 mg/kg, dissolved in 0.1 M cold citrate buffer; pH 4.5). The STZ-treated animals were allowed to drink 20% glucose solution for 24 h to overcome initial drug-induced hypoglycemic mortality. Three days after STZ injection, blood samples were collected through the tail vein and blood glucose levels were measured using a glucometer (OneTouch Horizon, LifeScan, Johnson & Johnson, CA, USA). Animals with blood glucose above 250 mg/dL were used for the study.

### 2.4. Experimental Procedures

Animals were divided into 4 groups of 10 animals each. The first group served as the control group. The second group contained STZ-induced diabetic rats and was left for 4 weeks untreated to induce early diabetic nephropathy [11]. The third and fourth groups were STZ-induced diabetic rats treated daily with low (1 mg/kg/day, p.o.) and high (10 mg/kg/day, p.o.) doses of carvedilol [12] by an oral gavage for 4 weeks. Carvedilol was suspended in 1% aqueous solution of carboxymethyl cellulose. All groups received equivalent volumes of the above-mentioned vehicles. The animals were placed in individual metabolic cages for 24 h to collect urine samples before the rats were killed. At the end of the experiment, each rat was weighed and then was sacrificed by cervical dislocation. Blood samples were collected and centrifuged at 3000 g for 10 min to obtain clear sera. The longitudinal section of the left kidney was excised from each animal for histological and immunohistochemical examination. The renal cortex of the rest of the kidneys was snap frozen in liquid nitrogen, stored at −80°C, and subsequently homogenized in cold potassium phosphate buffer (0.05 M, pH 7.4) for various biochemical analyses.

### 2.5. Biochemical Analysis

Using commercially available colorimetric kits, serum and urinary creatinine (Diamond Diagnostics, Egypt), 24 h urinary albumin (BioSystems, Spain), and serum glucose (Biodiagnostic, Egypt) as well as renal reduced glutathione (Biodiagnostic, Egypt) levels were quantified according to the manufacturers' guidelines. In addition, creatinine clearance, albumin/creatinine ratio, and kidney index were calculated. Renal tumor necrosis factor-*α* (TNF-*α*) assay was performed with rat TNF-*α* ELISA kit (RayBiotech, Inc., GA, USA) according to supplier's instructions. Renal cortex lipid peroxidation was determined as thiobarbituric acid reacting substance and is expressed as equivalents of malondialdehyde, using 1,1,3,3-tetramethoxypropane as standard [13]. Renal cortex nitric oxide level was measured as total nitrite/nitrate, the stable degradation products of nitric oxide, by reduction of nitrate into nitrite using copperized cadmium, followed by color development with Griess reagent in acidic medium [14].

### 2.6. Histological and Immunohistochemical Examination

Renal tissue samples were fixed in 10% neutral buffered formalin, embedded in paraffin, sectioned, and stained with hematoxylin and eosin for histological examination using light microscopy. The periodic acid-Schiff (PAS) stain was used for demonstration of glomerular basement membrane. In this method, the periodic acid oxidizes the carbon to carbon bond forming aldehydes that react to the fuchsin-sulfurous acid present in Schiff's reagent forming the magenta color.

For immunohistochemical detection of COX-2 expression, UltraVision ONE HRP polymer detection system (Thermo Fisher Scientific Inc./Lab Vision, Fremont, CA, USA) was used according to the manufacturer's protocol. Briefly, kidney sections were deparaffinized and rehydrated. Nonspecific binding of IgG was blocked using Ultra V block for 10 min at room temperature. The sections were then incubated with ready-to-use monoclonal COX-2 antibody for 1 h. After three washes, the sections were incubated for further 30 min with UltraVision ONE HRP polymer. Color reaction was developed by incubation with diaminobenzidine. The slides were then counterstained, dehydrated, and mounted.

### 2.7. Electron Microscopic Examination

For transmission electron microscopy, renal tissue samples were prefixed in 2% glutaraldehyde in phosphate buffer (pH 7.2). The specimens were then postfixed in 1% phosphate-buffered osmium tetroxide, dehydrated through ethanol and propylene oxide, and embedded in araldite. Semithin sections were stained with azure II and methylene blue. Ultrathin sections were obtained from the selected blocks, stained with uranyl acetate and lead citrate, examined, and then photographed.

### 2.8. Statistical Analysis

The data are expressed as means ± SEM. Statistical analysis was performed by one-way ANOVA followed by Tukey-Kramer postanalysis test for multiple comparisons with *P* < 0.05 being considered as statistically significant.

## 3. Results

### 3.1. Effects of Carvedilol on Renal Functions

**Figure 1:**
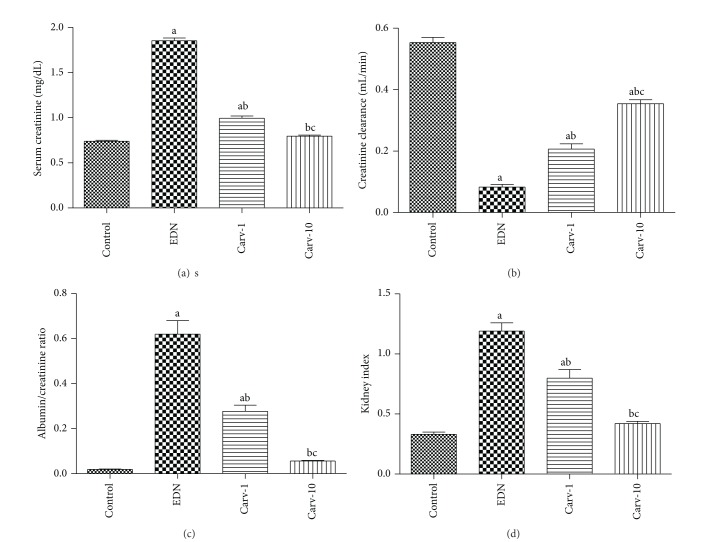
Effect of carvedilol (Carv; 1 and 10 mg/kg/day) on serum creatinine level (a), creatinine clearance (b), albumin/creatinine ratio (c), and kidney index (d) of streptozotocin-induced early diabetic nephropathy (EDN) in rats. Data are mean ± SEM of 10 rats. ^a,b,c^Significantly different from control, EDN, and Carv-1 groups, respectively, at *P* < 0.05.

Serum creatinine level, creatinine clearance, 24 h urinary albumin/creatinine ratio, and kidney index were assessed as markers of renal functions. Carvedilol treatments at both low and high doses significantly decrease serum creatinine level, 24 h urinary albumin/creatinine ratio, and kidney index with a concurrent increase in creatinine clearance compared to diabetic rats without treatment (Figures 1(a)–1(d)). Our preliminary experiments showed that carvedilol alone at both low and high doses did not alter renal function markers (data are not shown).

### 3.2. Effects of Carvedilol on Renal Malondialdehyde, Reduced Glutathione, and Nitrite/Nitrate Levels

**Figure 2:**
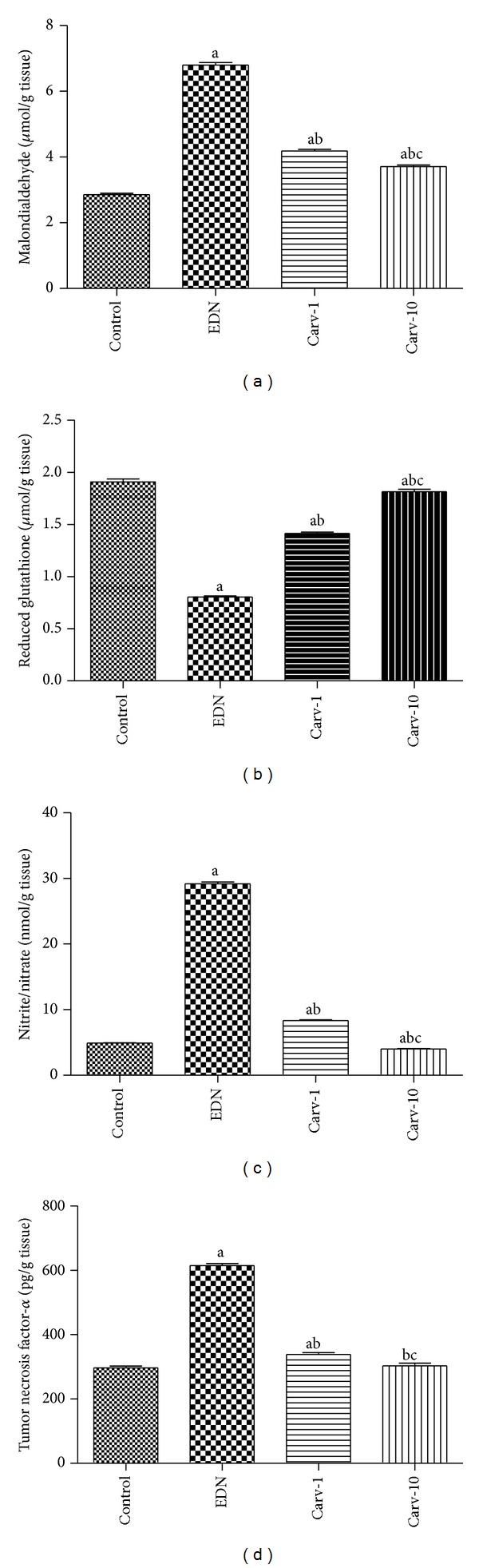
Effect of carvedilol (Carv; 1 and 10 mg/kg/day) on renal malondialdehyde (a), reduced glutathione (b), nitric oxide (as nitrite/nitrate; (c)), and tumor necrosis factor-*α* (d) levels of streptozotocin-induced early diabetic nephropathy (EDN) in rats. Data are mean ± SEM of 10 rats. ^a,b,c^Significantly different from control, EDN, and Carv-1 groups, respectively, at *P* < 0.05.

Oxidative stress was assessed through measuring renal malondialdehyde, reduced glutathione, and nitrite/nitrate levels. Renal malondialdehyde was evaluated as an indicator of renal lipid peroxidation and nitrite/nitrate as an indicator of renal nitric oxide level. Carvedilol treatment significantly suppressed both lipid peroxidation and the elevation of nitric oxide levels in comparison with diabetic untreated group (Figures 2(a) and 2(c)). On the other hand, carvedilol treatment caused significant increase in renal reduced glutathione level compared to diabetic rats without treatment (Figure 2(b)).

### 3.3. Effects of Carvedilol on TNF-*α* Level and COX-2 Expression

**Figure 3:**
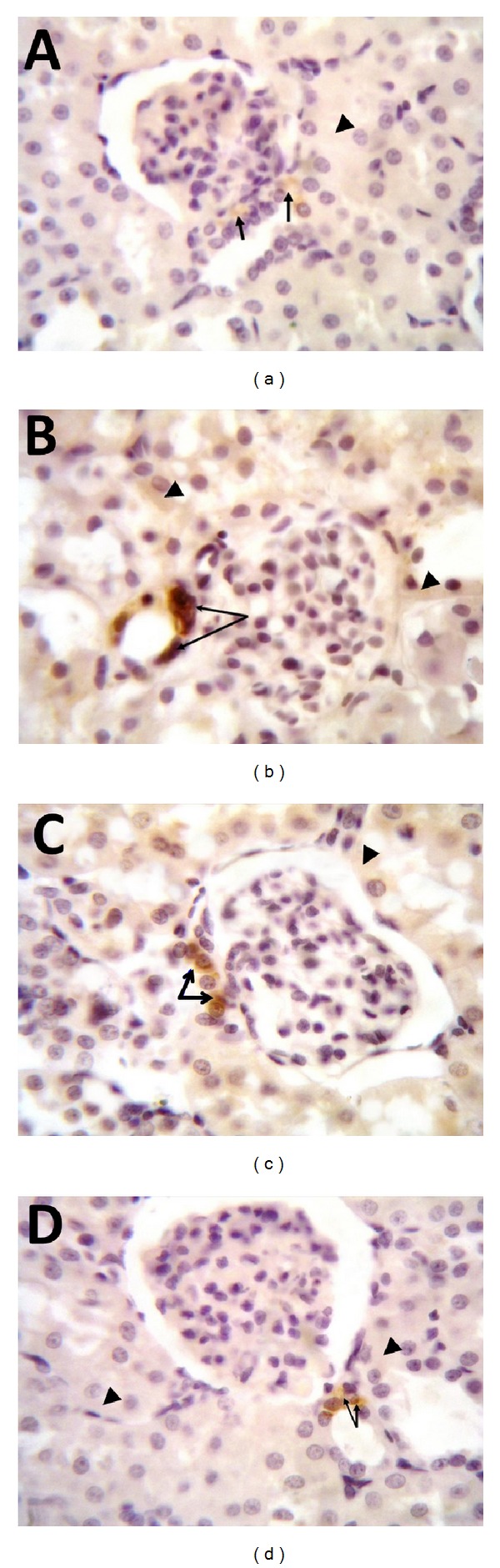
Effect of carvedilol (1 and 10 mg/kg/day) on immunohistochemical localization of cyclooxygenase-2 (COX-2) in the renal tissue of streptozotocin-induced early diabetic nephropathy in rats (100x). (a) Kidney tissue of nondiabetic rat; notice constitutional expression of COX-2 in the cytoplasm of macula densa cells (arrows) while tubular epithelial cells show negative signals (arrowhead). (b) Kidney tissue of diabetic rat showing increase of COX-2 expression in macula densa cells; notice that both the cytoplasm and the nucleus of macula densa cells are positive (arrows). Some of the tubular epithelial cells have cytoplasmic positive signals for COX-2 (arrowheads). (c) Kidney tissue after treatment with 1 mg carvedilol; COX-2 expression in the macula densa cells decreased and confined to the cytoplasm only (arrows) while tubular epithelial cells are still showing positive COX-2 expression (arrowhead). (d) Kidney tissue from diabetic rat treated with 10 mg carvedilol; notice normal level of COX-2 expression in the cytoplasm of macula densa cells (arrows) with negative expression in the tubular epithelial cells (arrowheads).

Both TNF-*α* level and COX-2 expression were increased in diabetic untreated rats compared to control group. On the other hand, these inflammatory mediators were decreased in diabetic rats with low or high dose carvedilol treatment compared to diabetic rats without treatment (Figures 2(d) and 3(a)–3(d)).

### 3.4. Effects of Carvedilol on Renal Histological Changes

**Figure 4:**
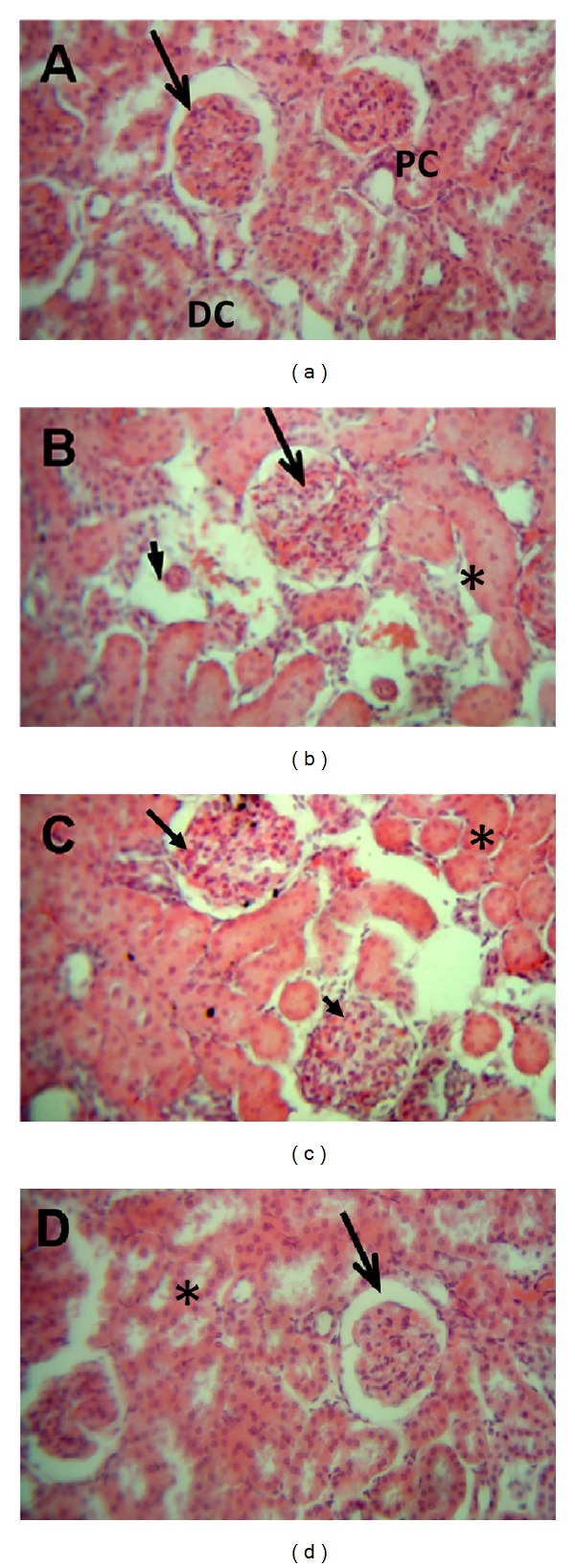
Effect of carvedilol (1 and 10 mg/kg/day) on kidney histopathological picture of streptozotocin-induced early diabetic nephropathy in rats (H&E 40x). (a) Kidney tissue of nondiabetic rat. Notice the normal appearance of the glomerular capillaries (arrow), with both proximal convoluted tubule (PC) and distal convoluted tubule (DC) showing normal epithelium. (b) Kidney tissue of diabetic rat; the glomerular capillaries are widened, irregular, and attached to Bowman's capsule (arrow). Furthermore, mesangial cell number was relatively higher in diabetic group. Some glomerular capillaries showing nodular sclerosis (arrowhead). Tubular epithelium is also affected (asterisk). (c) Kidney tissue after treatment with 1 mg carvedilol; the histological features are relatively improved compared to nontreated diabetic group. The glomerular capillaries retain their normal size and appearance (arrow), but mesangial cell number is still relatively higher than normal (arrowhead) and tubular epithelium is diminished (asterisk). (d) Kidney tissue from diabetic rat treated with 10 mg carvedilol; the histological features greatly improved and are nearly back to normal structure of glomerular capillaries (arrow) and tubular epithelium (asterisk).

**Figure 5:**
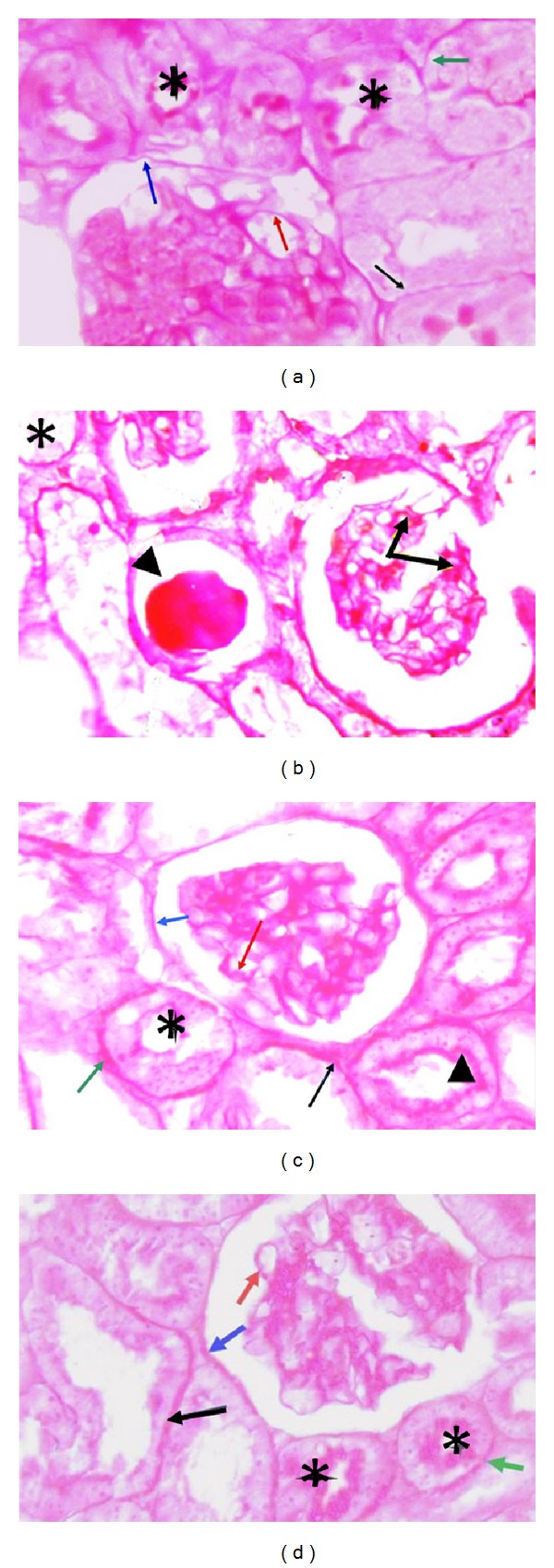
Effect of carvedilol (1 and 10 mg/kg/day) on glomerular basement membrane and Bowman's capsule of streptozotocin-induced early diabetic nephropathy in rats (PAS 100x). (a) Kidney tissue of nondiabetic rat showing normal thickness of glomerular basement membrane (red arrow), Bowman's capsule (blue arrow), proximal convoluted tubule (PC) (green arrow), and distal convoluted tubule (DT) (black arrow). Notice the brush border of PC (asterisks). (b) Kidney tissue of diabetic rat; notice increase in the thickness of glomerular basement membrane (arrows) and nodular substance stained positive with PAS (arrowhead). Notice loss of brush border of PC (asterisk). (c) Kidney tissue after treatment with 1 mg carvedilol; the histological features are relatively improved compared to nontreated diabetic group. Glomerular basement membrane is still thicker than normal (red arrow) and some of PC retained their brush border (arrowhead), while others did not (asterisk). Basement membrane of Bowman's capsule (blue arrow), PC (green arrow), and DC (black arrow) is still thick. (d) Kidney tissue from diabetic rat treated with 10 mg carvedilol; basement membrane of glomerular capillaries (red arrow), Bowman's capsule (blue arrow), PC (green arrow), and DC (black arrow) is of normal thickness. Notice normal brush border of PC (asterisks).

Histological changes were screened to support results of the markers of renal functions. Histopathological examination revealed that control group had normal appearance (Figures 4(a) and 5(a)). On the other hand, nontreated diabetic group demonstrated widened as well as irregular glomerular capillaries and relatively higher number of mesangial cells (Figure 4(b)) in association with increase in the thickness of the glomerular basement membrane (Figure 5(b)). Treatment with low dose carvedilol resulted in minor improvements (Figures 4(c) and 5(c)). On the other hand, the high dose carvedilol showed reversal of renal histopathological damage induced by STZ-induced early diabetic nephropathy, with normal structure of glomerular capillaries as well as tubular epithelium (Figures 4(d) and 5(d)). Improvement of renal histology in carvedilol treatment groups was not accompanied by any significant alteration in blood glucose level compared to diabetic rats not receiving carvedilol (data not shown).

### 3.5. Effects of Carvedilol on Electron Microscopic Analysis

**Figure 6:**
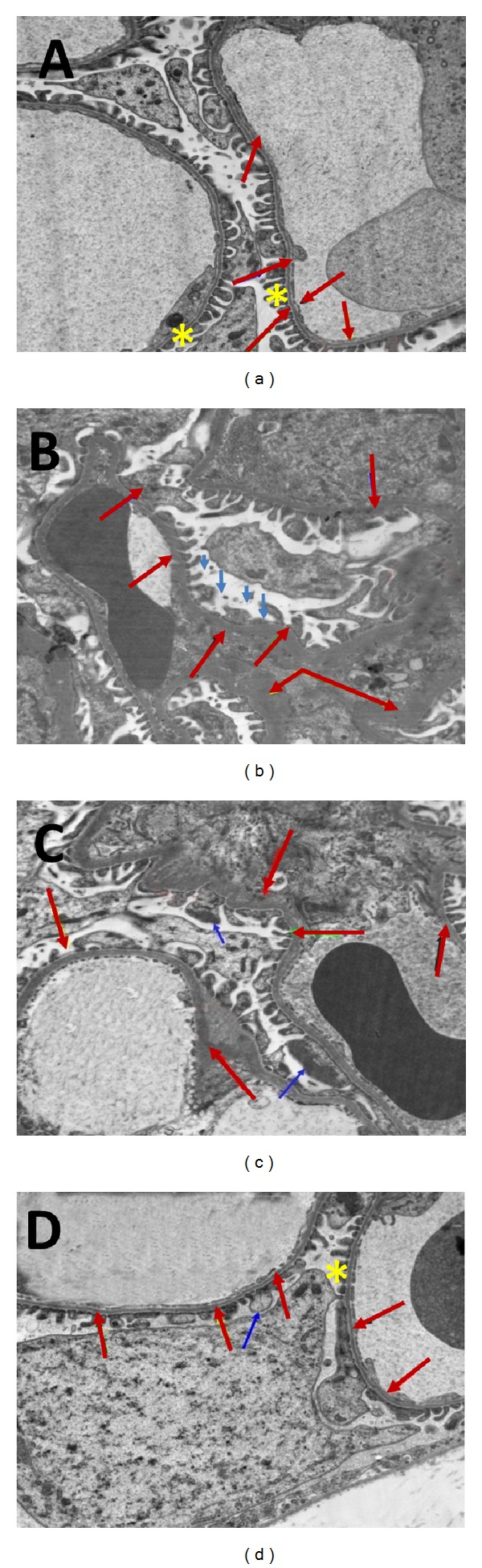
Transmission electron micrographs of rat renal tissue. (a) Kidney tissue of nondiabetic rat. Notice normal thickness and appearance of glomerular basement membrane (red arrows) and podocyte foot processes (asterisks). (b) Kidney tissue of diabetic rat showing thick and wrinkled glomerular basement membrane (red arrows). Effacement of podocyte foot processes (blue arrow). (c) Kidney tissue after treatment with 1 mg carvedilol. Notice little improvement in the thickness and course of the glomerular basement membrane (red arrows). Effacement of podocyte foot processes (blue arrows). (d) Kidney tissue from diabetic rat treated with 10 mg carvedilol; notice that the glomerular basement membrane is nearly of normal thickness and course (red arrows) and normal podocyte foot processes (asterisk) minimum effacement could be observed (blue arrow).

Electron microscopy revealed thick and wrinkled glomerular basement membrane and effacement of podocyte foot processes in diabetic group. These damages were markedly less severe particularly in the high dose carvedilol group when compared with the diabetic group (Figures 6(a)–6(d)).

## 4. Discussion

Carvedilol has vasodilatory, antioxidant, and anti-inflammatory properties, three actions that are likely to be effective in the prevention of early diabetic nephropathy. In the present study, carvedilol significantly decreased serum creatinine level and increased creatinine clearance. Consistent with these results, treatment with carvedilol significantly decreased plasma creatinine levels after ischemia-reperfusion injury [10] and cisplatin-induced nephrotoxicity [9]. Alternatively, carvedilol prevented reduction in the glomerular filtration rate in hypertensive patients with impaired kidney function [15]. On the other hand, in agreement with the current study, several previous studies denoted similar findings concerning the ability of carvedilol to decrease urinary albumin/creatinine ratio. For example, Bakris et al. [16] demonstrated that carvedilol attenuated the development of microalbuminuria in patients with type 2 diabetes and hypertension. Moreover, Jawa et al. [17] found that in type 2 diabetic African American patients with persistent microalbuminuria treatment with carvedilol, but not metoprolol, leads to significant reductions in urinary protein excretion.

Since oxidative stress plays an important role in the development of STZ-induced early diabetic nephropathy, several oxidative stress parameters were therefore assessed. On the other hand, Dugan et al. [18] revealed that mitochondrial-derived superoxide anion production is reduced in diabetes and plays an adaptive role in preserving renal function during hyperglycemia. In present study, the ability of carvedilol to increase renal reduced glutathione level is in line with the finding of Singh et al. [19] who found that carvedilol restored the depleted renal reduced glutathione level in ischemia-reperfusion renal injury. Reduced glutathione plays an important role in the antioxidant defense directly through scavenging reactive oxygen species and indirectly through functioning as a cofactor of antioxidant enzymes [20]. On the other hand, in accordance with the current study, Yasar et al. [7] reported that carvedilol protects against lipid peroxidation in ureteral obstruction-induced rat kidney injury. This inhibitory effect of carvedilol on lipid peroxidation could be explained to be secondary to its antioxidant activity through both direct radical scavenging and metal chelation [21]. In harmony with the present study, Yasar et al. [7] reported that carvedilol caused a reduction in renal nitric oxide level in injured rat kidney. Christo et al. [22] reported that nitric oxide has a role in the acute renal failure because of the free radical nature of the gasotransmitter nitric oxide that might contribute to tubular damage. In addition, nitric oxide increases renal injury through its reaction with superoxide radical and generation of a cytotoxic peroxynitrite [23], which could damage the tubular cells resulting in renal failure.

Inflammatory mediators including TNF-*α* and COX-2 play important roles in the pathogenesis of diabetic nephropathy. Accordingly, it is not surprising in our study to observe marked reduction of renal TNF-*α* level and COX-2 expression after carvedilol treatment. Our results concur with the finding of Arab and El-Sawalhi [6] who found that carvedilol lowered the release of the inflammatory cytokine TNF-*α* in sera and exudates of arthritic rats. Moreover, we show for the first time that administration of carvedilol significantly downregulated renal COX-2 expression. On the other hand, hyperglycemia is one of the crucial factors responsible for diabetic complications. However, in the present study carvedilol insignificantly altered blood glucose level compared to diabetic untreated rats. Thus, it appears that carvedilol protection is not secondary to glycemic control but related to its direct antioxidant and/or anti-inflammatory properties.

Podocyte injury is a typical characteristic in the development and/or progression of diabetic nephropathy. Additionally, glomerular basement membrane thickening has been considered an essential pathophysiological event in the disease [24]. Our data propose that the diabetic state caused significant structural changes in the glomerular basement membrane and podocytes, resulting in proteinuria. Reckelhoff et al. [25] found that diabetes induced glomerular basement membrane thickening. This irregular thickening may be due to increase in production and/or decrease in degradation of extracellular matrix proteins [26] and the degree of thickening has been shown to correlate with proteinuria [27]. On the other hand, Hoshi et al. [28] showed that progressive diabetic nephropathy is associated with podocyte injury in Zucker diabetic fatty rats and high glucose-induced podocyte stress in vitro causes damage in those cells. In the present study, we report for the first time that carvedilol treatment greatly improved the thickness of glomerular basement membranes and restored podocyte integrity. Lastly, the studied histopathology of carvedilol-treated rats indicates a status of structural integrity of the renal tissue and provides further support to the indicative mechanism of action of carvedilol. This result is in agreement with the finding of Hayashi et al. [10] who reported that the kidneys of carvedilol-treated rats showed significant decreases in histopathological changes associated with ischemia-reperfusion injury.

## 5. Conclusion

Taken together, the findings of the present study delineate a renal protective effect of carvedilol on the development of STZ-induced early diabetic nephropathy in rats. The protection afforded by carvedilol appears to stem from its antioxidant as well as anti-inflammatory activities and amelioration of podocyte injury.
